# 2-Benzyl-3-phenyl-1-(pyridin-2-yl)propan-1-one

**DOI:** 10.1107/S1600536812003686

**Published:** 2012-02-04

**Authors:** Muhammad Naveed Umar, Mohammad Shoaib, Seik Weng Ng

**Affiliations:** a School of Chemistry, University of Malakand, Khyber Pakhtunkhwa, Pakistan; b Department of Chemistry, University of Malaya, 50603 Kuala Lumpur, Malaysia, and Chemistry Department, Faculty of Science, King Abdulaziz University, PO Box 80203 Jeddah, Saudi Arabia

## Abstract

Mol­ecules of the title compound, C_21_H_19_NO, assume an approximate propellar shape, with the three aromatic rings being nearly perpendicularly aligned with respect to the plane formed by the C atoms that are connected to the methine C atom [dihedral angles: pyridyl 79.82 (4)°, phenyl 80.12 (3)° and phenyl 86.93 (3)°].

## Related literature

For background to fast aldol reactions, see: Nugent *et al.* (2010 ▶).
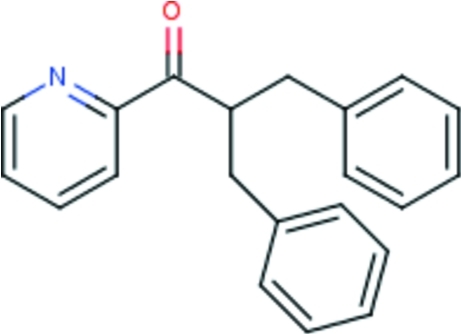


## Experimental

### 

#### Crystal data

C_21_H_19_NO
*M* *_r_* = 301.37
Monoclinic, 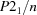

*a* = 15.1569 (3) Å
*b* = 5.6333 (1) Å
*c* = 19.5468 (4) Å
β = 109.295 (2)°
*V* = 1575.22 (5) Å^3^
*Z* = 4
Cu *K*α radiation
μ = 0.60 mm^−1^
*T* = 100 K
0.30 × 0.20 × 0.10 mm

#### Data collection

Agilent SuperNova Dual diffractometer with Atlas detector
Absorption correction: multi-scan (*CrysAlis PRO*; Agilent, 2011 ▶) *T*_min_ = 0.840, *T*_max_ = 0.942
25210 measured reflections
3299 independent reflections
3133 reflections with *I* > 2σ(*I*)
*R*_int_ = 0.034

#### Refinement

*R*[*F* ^2^ > 2σ(*F* ^2^)] = 0.036
*wR*(*F* ^2^) = 0.098
*S* = 1.04
3299 reflections
208 parameters
H-atom parameters constrained
Δρ_max_ = 0.26 e Å^−3^
Δρ_min_ = −0.21 e Å^−3^

### 

Data collection: *CrysAlis PRO* (Agilent, 2011 ▶); cell refinement: *CrysAlis PRO*; data reduction: *CrysAlis PRO*; program(s) used to solve structure: *SHELXS97* (Sheldrick, 2008 ▶); program(s) used to refine structure: *SHELXL97* (Sheldrick, 2008 ▶); molecular graphics: *X-SEED* (Barbour, 2001 ▶); software used to prepare material for publication: *publCIF* (Westrip, 2010 ▶).

## Supplementary Material

Crystal structure: contains datablock(s) global, I. DOI: 10.1107/S1600536812003686/bt5803sup1.cif

Structure factors: contains datablock(s) I. DOI: 10.1107/S1600536812003686/bt5803Isup2.hkl

Supplementary material file. DOI: 10.1107/S1600536812003686/bt5803Isup3.cml

Additional supplementary materials:  crystallographic information; 3D view; checkCIF report

## Figures and Tables

**Fig. 1.**
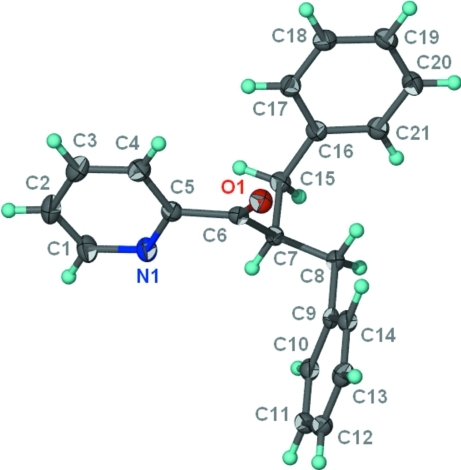
Anisotropic displacement ellipsoid plot (Barbour, 2001) of C21H19NO at the 70% probability level; hydrogen atoms are drawn as spheres of arbitrary radius.

## supplementary crystallographic information

### Comment

2-Benzyl-3-phenyl-1-(pyridin-2-yl)propan-1-one (Scheme I), in the optically active form, was synthesized for use in fast aldol condensations (Nugent *et al.*, 2010). The molecule assumes an approximate propellar shape (Fig. 1), with the three aromatic rings being nearly perpendicularly aligned at with respect to the plane formed by the C atoms that are connected to the methine C atom [dihedral angles: pyridyl 79.82 (4), phenyl 80.12 (3), phenyl 86.93 (3)°].

### Experimental

In a 250 ml flask was added sodium borohydride (4 equiv, 1.2 mg, 48 mmol) in anhydrous toluene (40 ml) followed by the addition of 18-crown-6 (0.1 equiv, 0.32 mg, 1.2 mmol), and acetyl pyridine (1 equiv, 1.35 ml, 12 mmol). Benzyl bromide (2.5 equiv, 3.6 ml, 30 mmol) was added. The reaction mixture was stirred at 323 K for 5 h under an inert atmosphere. The reaction was monitored by TLC and GC. The reaction was quenched by adding saturated ammonium chloride. The organic compound was extracted with ethyl acetate. The organic layer was dried over sodium sulfate and the solvent removed to give a yellow oil. This was submitted to flash chromatography and eluted with 5% ethyl acetate/hexane to give the desired ketone product (70% yield).

### Refinement

H-atoms were placed in calculated positions [C—H 0.95 to 0.99 Å, *U*_iso_(H) 1.2*U*_eq_(C)] and were included in the refinement in the riding model approximation.

### Crystal data

**Table:**

C_21_H_19_NO	*F*(000) = 640
*M**_r_* = 301.37	*D*_x_ = 1.271 Mg m^−^^3^
Monoclinic, *P*2_1_/*n*	Cu *K*α radiation, λ = 1.54184 Å
Hall symbol: -P 2yn	Cell parameters from 14175 reflections
*a* = 15.1569 (3) Å	θ = 3.1–76.1°
*b* = 5.6333 (1) Å	µ = 0.60 mm^−^^1^
*c* = 19.5468 (4) Å	*T* = 100 K
β = 109.295 (2)°	Block, colourless
*V* = 1575.22 (5) Å^3^	0.30 × 0.20 × 0.10 mm
*Z* = 4	

### Data collection

**Table:**

Agilent SuperNova Dual diffractometer with Atlas detector	3299 independent reflections
Radiation source: SuperNova (Cu) X-ray Source	3133 reflections with *I* > 2σ(*I*)
Mirror monochromator	*R*_int_ = 0.034
Detector resolution: 10.4041 pixels mm^-1^	θ_max_ = 76.3°, θ_min_ = 3.2°
ω scans	*h* = −19→19
Absorption correction: multi-scan (*CrysAlis PRO*; Agilent, 2011)	*k* = −7→5
*T*_min_ = 0.840, *T*_max_ = 0.942	*l* = −24→24
25210 measured reflections	

### Refinement

**Table:**

Refinement on *F*^2^	Primary atom site location: structure-invariant direct methods
Least-squares matrix: full	Secondary atom site location: difference Fourier map
*R*[*F*^2^ > 2σ(*F*^2^)] = 0.036	Hydrogen site location: inferred from neighbouring sites
*wR*(*F*^2^) = 0.098	H-atom parameters constrained
*S* = 1.04	*w* = 1/[σ^2^(*F*_o_^2^) + (0.0537*P*)^2^ + 0.5533*P*] where *P* = (*F*_o_^2^ + 2*F*_c_^2^)/3
3299 reflections	(Δ/σ)_max_ = 0.001
208 parameters	Δρ_max_ = 0.26 e Å^−^^3^
0 restraints	Δρ_min_ = −0.21 e Å^−^^3^

### Fractional atomic coordinates and isotropic or equivalent isotropic displacement parameters (Å^2^)

**Table:**

	*x*	*y*	*z*	*U*_iso_*/*U*_eq_	
O1	0.52050 (5)	0.37525 (13)	0.78840 (4)	0.01980 (17)	
N1	0.46273 (6)	0.74653 (15)	0.63746 (5)	0.0198 (2)	
C1	0.42604 (8)	0.7372 (2)	0.56524 (6)	0.0238 (2)	
H1	0.4365	0.8677	0.5381	0.029*	
C2	0.37344 (8)	0.5477 (2)	0.52749 (6)	0.0238 (2)	
H2	0.3482	0.5497	0.4761	0.029*	
C3	0.35863 (8)	0.3560 (2)	0.56657 (6)	0.0234 (2)	
H3	0.3218	0.2252	0.5425	0.028*	
C4	0.39850 (7)	0.35810 (19)	0.64159 (6)	0.0199 (2)	
H4	0.3908	0.2274	0.6698	0.024*	
C5	0.45003 (7)	0.55637 (17)	0.67445 (5)	0.0156 (2)	
C6	0.49749 (6)	0.56118 (17)	0.75561 (5)	0.0149 (2)	
C7	0.51069 (7)	0.80051 (17)	0.79253 (5)	0.0146 (2)	
H7	0.5282	0.9173	0.7607	0.018*	
C8	0.58912 (7)	0.79632 (18)	0.86598 (5)	0.0162 (2)	
H8A	0.5892	0.9489	0.8911	0.019*	
H8B	0.5763	0.6684	0.8961	0.019*	
C9	0.68460 (7)	0.75692 (17)	0.85936 (5)	0.0152 (2)	
C10	0.72381 (7)	0.92961 (18)	0.82695 (5)	0.0173 (2)	
H10	0.6902	1.0716	0.8093	0.021*	
C11	0.81153 (7)	0.89629 (19)	0.82020 (5)	0.0198 (2)	
H11	0.8372	1.0150	0.7978	0.024*	
C12	0.86173 (7)	0.68974 (19)	0.84614 (5)	0.0204 (2)	
H12	0.9218	0.6672	0.8418	0.024*	
C13	0.82336 (7)	0.51680 (18)	0.87838 (5)	0.0197 (2)	
H13	0.8573	0.3755	0.8963	0.024*	
C14	0.73529 (7)	0.54951 (18)	0.88463 (5)	0.0173 (2)	
H14	0.7094	0.4293	0.9063	0.021*	
C15	0.41552 (7)	0.87940 (17)	0.79944 (5)	0.0163 (2)	
H15A	0.4214	1.0450	0.8173	0.020*	
H15B	0.3674	0.8773	0.7507	0.020*	
C16	0.38254 (7)	0.72582 (17)	0.84959 (5)	0.0159 (2)	
C17	0.32976 (7)	0.52092 (18)	0.82459 (5)	0.0180 (2)	
H17	0.3112	0.4813	0.7746	0.022*	
C18	0.30400 (7)	0.37407 (18)	0.87213 (6)	0.0197 (2)	
H18	0.2680	0.2356	0.8544	0.024*	
C19	0.33078 (7)	0.42930 (19)	0.94531 (6)	0.0211 (2)	
H19	0.3140	0.3278	0.9778	0.025*	
C20	0.38230 (8)	0.6343 (2)	0.97072 (6)	0.0220 (2)	
H20	0.4005	0.6737	1.0207	0.026*	
C21	0.40717 (7)	0.78150 (18)	0.92306 (6)	0.0192 (2)	
H21	0.4415	0.9224	0.9408	0.023*	

### Atomic displacement parameters (Å^2^)

**Table:**

	*U*^11^	*U*^22^	*U*^33^	*U*^12^	*U*^13^	*U*^23^
O1	0.0225 (4)	0.0148 (4)	0.0208 (4)	0.0004 (3)	0.0055 (3)	0.0034 (3)
N1	0.0237 (4)	0.0177 (4)	0.0170 (4)	−0.0013 (3)	0.0055 (3)	0.0008 (3)
C1	0.0300 (6)	0.0227 (5)	0.0173 (5)	0.0001 (4)	0.0060 (4)	0.0025 (4)
C2	0.0255 (5)	0.0263 (6)	0.0161 (5)	0.0040 (4)	0.0021 (4)	−0.0016 (4)
C3	0.0218 (5)	0.0211 (5)	0.0235 (5)	−0.0015 (4)	0.0024 (4)	−0.0054 (4)
C4	0.0196 (5)	0.0169 (5)	0.0225 (5)	−0.0012 (4)	0.0061 (4)	−0.0005 (4)
C5	0.0146 (4)	0.0152 (5)	0.0172 (5)	0.0017 (3)	0.0054 (4)	−0.0002 (4)
C6	0.0127 (4)	0.0153 (5)	0.0177 (5)	−0.0008 (3)	0.0062 (4)	0.0008 (4)
C7	0.0156 (4)	0.0140 (4)	0.0138 (4)	−0.0001 (3)	0.0043 (4)	0.0013 (3)
C8	0.0165 (5)	0.0179 (5)	0.0138 (4)	−0.0009 (4)	0.0043 (4)	−0.0007 (3)
C9	0.0163 (5)	0.0173 (5)	0.0110 (4)	−0.0024 (4)	0.0029 (3)	−0.0026 (3)
C10	0.0196 (5)	0.0168 (5)	0.0141 (4)	−0.0011 (4)	0.0037 (4)	−0.0001 (4)
C11	0.0217 (5)	0.0223 (5)	0.0166 (5)	−0.0053 (4)	0.0077 (4)	−0.0015 (4)
C12	0.0172 (5)	0.0250 (5)	0.0196 (5)	−0.0020 (4)	0.0071 (4)	−0.0057 (4)
C13	0.0200 (5)	0.0181 (5)	0.0191 (5)	0.0013 (4)	0.0040 (4)	−0.0027 (4)
C14	0.0197 (5)	0.0169 (5)	0.0147 (4)	−0.0024 (4)	0.0048 (4)	−0.0007 (4)
C15	0.0166 (5)	0.0151 (5)	0.0171 (5)	0.0019 (3)	0.0052 (4)	0.0018 (3)
C16	0.0138 (4)	0.0157 (5)	0.0185 (5)	0.0030 (3)	0.0056 (4)	0.0016 (4)
C17	0.0164 (4)	0.0196 (5)	0.0169 (4)	0.0003 (4)	0.0040 (4)	−0.0003 (4)
C18	0.0169 (5)	0.0183 (5)	0.0230 (5)	−0.0018 (4)	0.0054 (4)	0.0007 (4)
C19	0.0203 (5)	0.0226 (5)	0.0219 (5)	0.0003 (4)	0.0092 (4)	0.0048 (4)
C20	0.0237 (5)	0.0262 (5)	0.0174 (5)	−0.0005 (4)	0.0084 (4)	−0.0007 (4)
C21	0.0197 (5)	0.0177 (5)	0.0204 (5)	−0.0012 (4)	0.0071 (4)	−0.0023 (4)

### Geometric parameters (Å, º)

**Table:**

O1—C6	1.2172 (12)	C10—H10	0.9500
N1—C1	1.3365 (14)	C11—C12	1.3910 (15)
N1—C5	1.3414 (13)	C11—H11	0.9500
C1—C2	1.3897 (16)	C12—C13	1.3881 (15)
C1—H1	0.9500	C12—H12	0.9500
C2—C3	1.3829 (16)	C13—C14	1.3922 (14)
C2—H2	0.9500	C13—H13	0.9500
C3—C4	1.3893 (15)	C14—H14	0.9500
C3—H3	0.9500	C15—C16	1.5111 (13)
C4—C5	1.3922 (14)	C15—H15A	0.9900
C4—H4	0.9500	C15—H15B	0.9900
C5—C6	1.5103 (13)	C16—C21	1.3950 (14)
C6—C7	1.5110 (13)	C16—C17	1.3974 (14)
C7—C8	1.5320 (13)	C17—C18	1.3928 (14)
C7—C15	1.5573 (13)	C17—H17	0.9500
C7—H7	1.0000	C18—C19	1.3869 (15)
C8—C9	1.5113 (13)	C18—H18	0.9500
C8—H8A	0.9900	C19—C20	1.3905 (15)
C8—H8B	0.9900	C19—H19	0.9500
C9—C10	1.3963 (14)	C20—C21	1.3892 (15)
C9—C14	1.3960 (14)	C20—H20	0.9500
C10—C11	1.3916 (14)	C21—H21	0.9500
			
C1—N1—C5	117.01 (9)	C12—C11—C10	120.20 (9)
N1—C1—C2	123.81 (10)	C12—C11—H11	119.9
N1—C1—H1	118.1	C10—C11—H11	119.9
C2—C1—H1	118.1	C13—C12—C11	119.46 (9)
C3—C2—C1	118.42 (10)	C13—C12—H12	120.3
C3—C2—H2	120.8	C11—C12—H12	120.3
C1—C2—H2	120.8	C12—C13—C14	120.31 (10)
C2—C3—C4	118.89 (10)	C12—C13—H13	119.8
C2—C3—H3	120.6	C14—C13—H13	119.8
C4—C3—H3	120.6	C13—C14—C9	120.75 (9)
C3—C4—C5	118.41 (10)	C13—C14—H14	119.6
C3—C4—H4	120.8	C9—C14—H14	119.6
C5—C4—H4	120.8	C16—C15—C7	114.03 (8)
N1—C5—C4	123.40 (9)	C16—C15—H15A	108.7
N1—C5—C6	116.65 (8)	C7—C15—H15A	108.7
C4—C5—C6	119.92 (9)	C16—C15—H15B	108.7
O1—C6—C5	119.46 (9)	C7—C15—H15B	108.7
O1—C6—C7	123.14 (8)	H15A—C15—H15B	107.6
C5—C6—C7	117.36 (8)	C21—C16—C17	118.25 (9)
C6—C7—C8	111.91 (8)	C21—C16—C15	120.38 (9)
C6—C7—C15	108.41 (8)	C17—C16—C15	121.33 (9)
C8—C7—C15	112.17 (8)	C18—C17—C16	120.77 (9)
C6—C7—H7	108.1	C18—C17—H17	119.6
C8—C7—H7	108.1	C16—C17—H17	119.6
C15—C7—H7	108.1	C19—C18—C17	120.25 (10)
C9—C8—C7	112.99 (8)	C19—C18—H18	119.9
C9—C8—H8A	109.0	C17—C18—H18	119.9
C7—C8—H8A	109.0	C18—C19—C20	119.55 (10)
C9—C8—H8B	109.0	C18—C19—H19	120.2
C7—C8—H8B	109.0	C20—C19—H19	120.2
H8A—C8—H8B	107.8	C21—C20—C19	120.06 (10)
C10—C9—C14	118.47 (9)	C21—C20—H20	120.0
C10—C9—C8	119.98 (9)	C19—C20—H20	120.0
C14—C9—C8	121.55 (9)	C20—C21—C16	121.10 (10)
C11—C10—C9	120.82 (9)	C20—C21—H21	119.5
C11—C10—H10	119.6	C16—C21—H21	119.5
C9—C10—H10	119.6		
			
C5—N1—C1—C2	2.48 (16)	C14—C9—C10—C11	−0.23 (14)
N1—C1—C2—C3	−0.61 (17)	C8—C9—C10—C11	−179.84 (9)
C1—C2—C3—C4	−1.43 (16)	C9—C10—C11—C12	−0.31 (15)
C2—C3—C4—C5	1.50 (16)	C10—C11—C12—C13	0.38 (15)
C1—N1—C5—C4	−2.38 (15)	C11—C12—C13—C14	0.09 (15)
C1—N1—C5—C6	175.79 (9)	C12—C13—C14—C9	−0.64 (15)
C3—C4—C5—N1	0.44 (15)	C10—C9—C14—C13	0.70 (14)
C3—C4—C5—C6	−177.67 (9)	C8—C9—C14—C13	−179.69 (9)
N1—C5—C6—O1	−151.38 (9)	C6—C7—C15—C16	66.21 (10)
C4—C5—C6—O1	26.85 (13)	C8—C7—C15—C16	−57.86 (11)
N1—C5—C6—C7	30.88 (12)	C7—C15—C16—C21	90.50 (11)
C4—C5—C6—C7	−150.88 (9)	C7—C15—C16—C17	−87.20 (11)
O1—C6—C7—C8	24.09 (13)	C21—C16—C17—C18	−1.18 (15)
C5—C6—C7—C8	−158.27 (8)	C15—C16—C17—C18	176.56 (9)
O1—C6—C7—C15	−100.14 (10)	C16—C17—C18—C19	−0.18 (15)
C5—C6—C7—C15	77.51 (10)	C17—C18—C19—C20	0.99 (16)
C6—C7—C8—C9	66.67 (10)	C18—C19—C20—C21	−0.42 (16)
C15—C7—C8—C9	−171.23 (8)	C19—C20—C21—C16	−0.98 (16)
C7—C8—C9—C10	66.63 (11)	C17—C16—C21—C20	1.76 (15)
C7—C8—C9—C14	−112.97 (10)	C15—C16—C21—C20	−176.00 (9)
